# Hyperthyroidism and the Risk of Cardiac Arrhythmias: A Narrative Review

**DOI:** 10.7759/cureus.24378

**Published:** 2022-04-22

**Authors:** Mahlika Ahmad, Sanjana Reddy, Zineb Barkhane, Jalal Elmadi, Lakshmi Satish Kumar, Lakshmi Sree Pugalenthi

**Affiliations:** 1 Internal Medicine, Ziauddin University, Karachi, PAK; 2 Medicine, Bogomolets National Medical University, Kiev, UKR; 3 Research, Faculty of Medicine and Pharmacy, Université Hassan II de Casablanca, Casablanca, MAR; 4 Faculty of Medical Sciences, Universidad Nacional Autónoma de Honduras, Tegucigalpa, HND; 5 Medicine, University of Perpetual Help System DALTA (Daisy Antonio Laperal Tamayo), Manila, PHL; 6 Research, Ago Medical and Educational Center Bicol Christian College of Medicine (AMEC BCCM), Legazpi City, PHL

**Keywords:** management of thyrotoxicosis, cardiac arrhythmias, ventricular arrhythmias, heart rate, overt hyperthyroidism, subclinical hyperthyroidism, thyrotoxicosis, sinus tachycardia, atrial fibrillation, hyperthyroidism

## Abstract

Hyperthyroidism directly affects the cardiovascular system, altering the heart's normal function and leading to high cardiovascular mortality. Excess thyroid hormones are associated with significantly increased risk and prevalence of cardiac arrhythmias, particularly atrial fibrillation (AF). This article reviewed the hemodynamic changes and the risk of cardiac arrhythmias, including atrial and ventricular arrhythmias associated with hyperthyroidism. It has also discussed the multi-level pathophysiology of thyrotoxic AF, sinus tachycardia, and different treatment modalities such as anti-thyroid drugs, beta-blockers, and the role of cardioversion and catheter ablation. This article has explored different studies that have concluded that AF and sinus tachycardia are the most common arrhythmias associated with thyrotoxicosis.

## Introduction and background

Hyperthyroidism, also known as thyrotoxicosis, results from increased thyroid levels in the body. It can develop due to increased thyroid hormone synthesis or increased release from the gland [1]. In a study conducted by National Health and Nutrition Examination Survey (NHANES III), the incidence of hyperthyroidism was reported as 1.3% in the United States of America (USA) by measuring the serum thyroid-stimulating hormone (TSH), thyroxin (T4), and thyroid antibodies [2]. In addition, hyperthyroidism was more prevalent in females (1.37%) as compared to the male gender (0.3%) [3]. The most sensitive and specific test for diagnosing hyperthyroidism or suspected thyrotoxicosis, also used for screening purposes, is TSH levels in the blood. [1]. Hyperthyroidism is classified into overt and subclinical subtypes. In overt hyperthyroidism, serum TSH levels are suppressed with serum levels of T3, and free T4 is elevated. In contrast, the subclinical type is a low or undetectable blood TSH level with T3 and free T4 within the normal reference range [1,4]. Graves' disease is the most common cause of thyrotoxicosis. At the same time, thyroiditis, toxic multinodular goiter, toxic adenomas, and side effects of certain medications are fewer common disorders resulting in this condition [5]. Hyperthyroidism can present with various symptoms, ranging from asymptomatic to a severe hyperdynamic response [6]. Some of the profound effects of elevated thyroid hormone levels are on the cardiovascular system, out of which sinus tachycardia and atrial fibrillation (AF) are the most common manifestations of thyrotoxic heart disease. A study conducted in France reported an increased occurrence of cardiac dysrhythmias such as AF and other complications in the elderly (33.9%) as compared to younger patients with thyroid disease (11.3%) [7]. This review aims to elaborate on the association of hyperthyroidism or thyrotoxicosis with cardiac arrhythmias such as atrial premature contractions, AF, ventricular premature contractions, and malignant ventricular arrhythmias, including ventricular tachycardia and ventricular fibrillation [8]. 

## Review

Effect of thyroid hormone on the cardiac musculature

Thyroid hormone has a significant hyperdynamic response in the body, affecting the heart directly and indirectly. Several studies conducted over the last few decades have provided critical insights into the mechanisms underlying the hyperdynamic cardio-circulatory state in hyperthyroid patients, indicating that it most likely results from the combined effects of thyroid hormone on specific molecular pathways in the heart and vasculature, at both the genomic and non-genomic levels [9]. Direct T3 effects result from T3 action on the heart and are mediated by nuclear or extranuclear mechanisms. Nuclear T3 mechanisms are mediated by thyroid hormone bound to thyroid responsive elements (TREs) that regulate cardiac gene expression by altering the production of particular messenger RNA (mRNA) and translated proteins and creating tissue-specific responses that have a direct impact on cardiac myocytes. On the other hand, the extranuclear T3 effects, which occur independently of nuclear T3 receptor binding, are implicated in structural and regulatory proteins. Long-term T3 exposure increases cardiac protein synthesis, mainly regulating the transport of amino acids, carbohydrates, and calcium (Ca^+2^) through the cell membrane leading to ventricular hypertrophy and dysfunction [10,11].

The non-genomic activity includes rapid changes in the plasma membrane and cytoplasmic organelles of cardiac myocytes. The thyroid hormone exerts its effects on myocytes by upregulating the alpha myosin heavy chain and, at the same time, downregulating the beta (b) myosin chain leading to an increase in relative tissue level of sarcoplasmic reticulum calcium ATPase (SERCA). In contrast, levels of phospholamban were decreased, making the relative ratio of phospholamban and Ca^+2^ ATPase lower in hyperthyroid patients compared to hypothyroid patients enhancing myocardial contractility [11]. Thyroid hormone also affects ion channels such as sodium-potassium ATPase (Na^+^/K^+^ ATPase), Na^+^/Ca^+2^ exchangers (NCX), and specific voltage-gated K^+^ channels, influencing cardiac and vascular functioning [12,13], downregulating the adenylyl cyclase catalytic subunits and thyroid hormone alpha-1 receptor. All these changes collectively inhibit myocardial relaxation [11]. Furthermore, thyroid hormone causes significant hyperdynamic changes, by immediate utilization of oxygen in the peripheral vascular system, increased generation of metabolic end products, and relaxation of arterial smooth muscle fibers by thyroid hormone, which all contribute to peripheral vasodilation.

**Figure 1:**
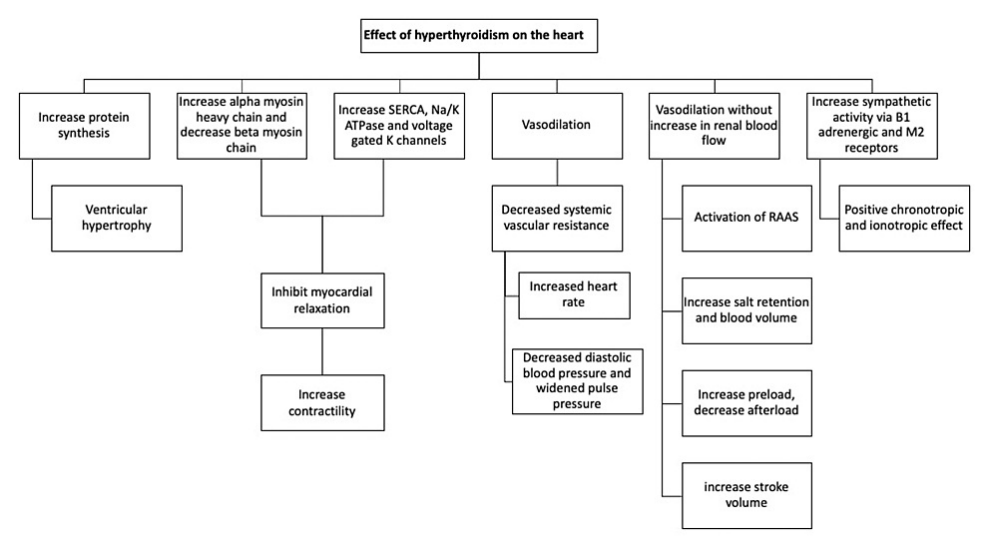
Effect of thyroid hormone on the heart SERCA: sarcoplasmic reticulum calcium ATPase; Na: sodium; K: potassium; RAAS: renin-angiotensin aldosterone system; B1: beta1; M2 receptors: muscarinic-2 receptors

This decrease in peripheral vascular resistance (PVR) is key to all thyroid hormone-induced hemodynamic alterations. Thus, a decrease in PVR causes an increase in heart rate, a selective increase in blood flow to particular organs (skin, skeletal muscles, heart), and a decrease in diastolic pressure, resulting in widening pulse pressure [14]. Vasodilation without an increase in renal blood flow reduces renal perfusion. It activates the renin-angiotensin-aldosterone system (RAAS), resulting in salt retention and increased blood volume, increasing the preload and decreasing the afterload, leading to a significant increase in the stroke volume [15,16]. Increased thyroid hormone affects the beta1-adrenergic and M2-muscarinic receptors of the heart, causing an enhanced sympathetic activity, tachycardia, and shortened atrial refractory time in hyperthyroid individuals. Hence, thyroid hormone has positive inotropic and chronotropic effects on the heart, leading to an increased adrenergic sensitivity that accounts for elevated heart rate and contractility [15]. In contrast, a study reported that the overall susceptibility of cardiac myocytes to adrenergic stimulation stays unaltered. This is corroborated by the fact that administering a beta-adrenergic receptor antagonist to hyperthyroid individuals reduces their heart rate but has no impact on systolic or diastolic contraction, suggesting that the positive inotropic effect is independent of adrenergic signaling pathways [11]. Hyperthyroidism hemodynamic changes reduce the myocardial contractile reserve, preventing further increase in ejection fraction and cardiac output during exercise, making the person predisposed to heart failure (HF) (Figure 1) [15-19].

Current evidence of thyrotoxicosis and cardiac arrhythmias

Atrial Fibrillation

AF has been the most prevalent clinically significant arrhythmias associated with thyrotoxicosis which presents in 28% of patients [8]. Overt and subclinical hyperthyroidism are well-known independent risk factors for AF [20]. Frost et al. conducted a population-based study in Denmark to examine the risk of AF among patients with hyperthyroidism aged 20 to 89 years for 20 years. Among these patients, 8.3% were diagnosed as having AF within ± 30 days from the day of diagnosis of hyperthyroidism. Moreover, other risk factors for AF were identified, including male gender, advancing age, ischemic heart disease (IHD), congestive heart failure (CHF), and heart valve diseases [21]. Sawin et al. conducted an original cohort study on men and women aged 60 years or older that reported a threefold increase in the risk of cardiac arrhythmias in people with low TSH levels. The incidence of AF was 28% in subjects with low TSH levels compared to 11% among the ones with normal values of TSH [22]. In a Rotterdam study, the risk of AF was associated with TSH levels; people with a high normal function of thyroid hormone were at an increased risk of developing AF [20]. Moreover, Iwasaki et al. reported that individuals with AF had biochemically more severe hyperthyroidism (higher T3 and T4 levels) [23]. An increased risk of arterial embolism was reported in patients with thyrotoxicosis and AF [24]. A cross-sectional study done by Auer et al. compared subjects with overt and subclinical hyperthyroidism and the prevalence of AF, which showed the prevalence of AF was slightly higher in patients with overt hyperthyroidism (13.8%) as compared to subclinical hyperthyroidism (12.7%); the prevalence of AF was 2.3% in euthyroid individuals, which was the control group. Overall, decreased TSH levels were associated with a more than five-fold higher incidence of AF with no significant differences between overt and subclinical hyperthyroidism [25].

AF occurs when the common sinus mechanism is suppressed or replaced by a diffuse and chaotic pattern of electrical activity in the atria [26]. It can be maintained by re-entry and/or rapid focal ectopic firing [27]. The mechanism that maintains AF is commonly referred to as the driver. Irregular atrial discharge characteristic of AF may be caused by an irregular atrial response to a quickly regularly discharged firing driver caused by either local ectopic firing or a single localized re-entry circuit. Alternatively, fibrillatory activity might be induced by numerous functioning re-entry circuits that fluctuate in time and place. Electric remodeling modifies ion channel expression and function, promoting AF. Because Ca^2+ ^enters atrial cells with each action potential, rapid atrial rates increase Ca^2+^ loading and initiate autoprotective mechanisms that reduce Ca^2+^ entry: Ca^2+^ current inactivation and L-type calcium current (ICa,L) downregulation (which directly reduces Ca^2+^ entry), and inward rectifier K^+^ current enhancement both (I_K1_) and constitutive acetylcholine-dependent current (I_K,AChc_), which decreases Ca^2+^ loading by reducing action potential duration (APD). These alterations stabilize atrial re-entry rotors by lowering APD, boosting AF vulnerability and sustainability. Furthermore, changes in Ca^2+^ processing enhance diastolic Ca^2+^ release and ectopic activity [27-30]. In a study by Hu et al., they experimented on hyperthyroid mice myocytes, evaluating the mRNA and protein expression levels of K^+^ channel alpha subunits in the left and right atria. As a result, it is hypothesized that decreased interatrial APD may increase the propagation of abnormal activity in hyperthyroid conditions and offer a substrate for atrial arrhythmias like AF (Table 1) [31].

**Table 1:** Mechanisms of atrial fibrillation in thyrotoxicosis APD: action potential duration

Mechanisms of AF in thyrotoxicosis
The APD is shortened in both the right and left atrial myocytes.
Protein expression levels of Kv1.5 and Kv2.1 are significantly higher in both atria.
The influence of hyperthyroidism on APD and delayed rectifier K^+^ currents are more prominent in the right atrium than in the left, minimizing the interatrial APD difference.
Thyroid hormone causes amplified automaticity and triggering activity that increases the arrhythmogenic potential of pulmonary veins in hyperthyroidism.

Moreover, Watanabe et al. revealed that ICa,L mRNA expression was decreased in hyperthyroid compared to euthyroid myocytes [32]. Another mechanism behind the initiation of paroxysmal AF is the arrhythmogenic nature of pulmonary vein (PV) cardiomyocytes. Chen et al. examined the ionic currents in PV cardiomyocytes, concluding a higher incidence of early afterdepolarization (46% vs. 0%, p<0.0001). Furthermore, hyperthyroid PV cardiomyocytes had faster beating rates (1.82 +/- 0.13 Hz vs. 1.03 +/- 0.15 Hz, p < 0.005) and an overall more significant density of transient outward, steady-state outward, and transient inward currents [33]. Additionally, shortening the atrial refractory period has been another primary mechanism for increased risk of AF in hyperthyroidism, reflecting a strong association with increased supraventricular ectopic activity in individuals with normal hearts. Wustmann et al. conducted a prospective trial on 28 patients with a mean age of 43+/-11 years who were newly diagnosed and with untreated hyperthyroidism. The patients were followed up over 16+/-6 months and six months after stabilizing serum TSH levels. They evaluated the activity of abnormal supra ventricular electrical depolarisation at baseline and follow-up after the TSH levels were normal. The study concluded that the incidence of supraventricular premature depolarization and the number of episodes of supraventricular tachycardia decreased significantly after serum TSH levels were normalized [34].

Sinus tachycardia

Hyperthyroidism causes symptoms similar to catecholamine excess, including palpitations due to sinus tachycardia. Recent studies have confirmed that these symptoms are not due to increased epinephrine and norepinephrine [35], as epinephrine and norepinephrine levels are normal in hyperthyroidism patients [36]. However, a study conducted to evaluate the effect of atropine on tachycardia in hyperthyroidism suggested that in thyrotoxicosis patients, resting tachycardia is caused by a combination of T4 direct impact on the heart by increasing activity of the sinoatrial (SA) node and impairment of vagal regulation of heart rate [37]. Hyperthyroidism is characterized by a sympathovagal imbalance, with enhanced sympathetic and reduced vagal regulation of the heart rhythm. These autonomic dysfunctions can be recognized concurrently by spectral analysis of heart rate variability (HRV) patients, and spectral HRV parameters may represent clinical severity in hyperthyroid patients [38]. HRV is a non-invasive physiological marker for assessing the autonomic nervous system, reflecting beat to beat variability in R-R intervals indicating imbalances between the parasympathetic and sympathetic activity of the autonomic nervous system (ANS) [39-41] and is a valuable tool to monitor the cardiovascular risk and significant clinical consequences and help in the therapy choice [24]. This was reported by the study conducted by Burggraaf et al. that demonstrated an increased heart rate, reduced R-R interval variability and increased 24-hour urinary excretion of catecholamines [42].

Moreover, non-selective beta-blockade reduced the heart rate but did not affect the HRV. Another study by Valcavi et al. concluded that total autonomic blockade in thyrotoxic patients restored electrophysiological parameters to values comparable to those seen in euthyroid patients. Furthermore, the intrinsic activity of the node is elevated in thyrotoxic individuals. This appears to be a direct result of thyroid hormone excess rather than an outcome of external forces placed on SA node activity by the ANS [43]. Thyrotoxic individuals also have abnormalities in left ventricular shape and elevated echocardiographic markers of myocardial contractility, whereas patients with subclinical hyperthyroidism only have an increased velocity of left ventricular relaxation. Cardiac parasympathetic withdrawal is observed in overt and subclinical hyperthyroid patients [44].

Tudoran et al. conducted a study in 2019 to determine HRV and heart rate turbulence in 113 women with overt hyperthyroidism without any cardiovascular risk factors over four years by 24-hour Holter monitoring. They were divided into three groups depending on the severity of the disease. HRV measures showed an R-R interval (0.74 ± 0.10 vs. 0.91 ± 0.11; 0.006) and heart rate (82.91 ± 10.99 vs. 67.04 ± 6.80; 0.006) significantly higher in patients with thyrotoxicosis. Sinus tachycardia was reported in 56.52% of patients with mild to moderate hyperthyroidism, 76.47% of patients with severe hyperthyroidism, and 54.54% of women with chronic forms [45]. When compared to controls (24.13%), patients with hyperthyroidism had a greater incidence of premature supraventricular contractions (41.3%, 61.76% , 48.48% respectively) [45]. Another study reported that patients with subclinical hyperthyroidism showed similar changes in heart rate and variability compared to control groups [46]. Subclinical thyroid disease dramatically altered heart rate profiles during rest, activity, and recovery [47].

Similarly, in a cohort study conducted in sub-African female patients, heart rate was significantly higher in patients with thyrotoxicosis than in the control group. In terms of arrhythmias, sinus tachycardia was a common finding in those with hyperthyroidism but only occurred in 20.68% of controls [48]. In a study done to analyze the arrhythmia profile and heart rate by 24-hour Holter monitoring in 37 hyperthyroid patients before and after the anti-thyroid therapy, the data was compared with control subjects free from cardiovascular disease. The hyperthyroid patients showed frequent supraventricular arrhythmias that could be reversed during anti-thyroid treatment. However, ventricular arrhythmias were infrequent and remained unchanged after treatment [13]. Improved diagnosis of supraventricular dysrhythmias and therapeutic intervention (e.g., anticoagulants, antiarrhythmics) may enhance long-term vascular prognosis, although their function in extensive therapeutic trials is yet to be demonstrated [49]. In contrast to supraventricular arrhythmias, ventricular arrhythmias occurred at a rate comparable to the general population. Furthermore, before and after anti-thyroid treatment, the incidence of ventricular arrhythmias in thyrotoxic individuals stays constant. Ventricular tachycardia and ventricular fibrillation are uncommon in thyrotoxicosis patients and usually occur in individuals with severe HF or accompanying cardiac illness [49,50].

Risk of cardiovascular disease and mortality 

The early screening and treatment of thyroid dysfunction in patients with arrhythmias are essential because the long-term prognosis could be improved with adequate management of thyroid dysfunction [8]. A single measurement of low TSH levels in people aged 60 years and above was related to an increased risk of death from any cause, especially from circulatory and cardiovascular disorders [51]. Consistently, reliable evidence exists for increased cardiovascular morbidity in overt hyperthyroidism and is associated with predictors of cardiovascular mortality like ventricular hypertrophy, ventricular dysfunction, and AF. As for subclinical hyperthyroidism, evidence is conclusive only concerning an up to 5.2-fold elevated risk for AF [52]. In overt hyperthyroidism, a 1.7-fold increased risk of cardiovascular disease and a 1.7-fold increased risk of mortality was demonstrated [52]. Sub-clinical hyperthyroidism is not associated with CHF or mortality from other cardiovascular causes [53]. Another study demonstrated that subclinical hyperthyroidism significantly increased the risk of cardiovascular disease for the general population and cardiovascular and all-cause mortality for individuals with other morbidities [54]. Brandt et al. conducted a meta-analysis including eight studies that fulfilled the inclusion criteria, suggesting a 20% increase in overall mortality in patients with hyperthyroidism [55]. Long-term follow-up studies have demonstrated an increase in cardiovascular and cerebrovascular disease mortality in those with a history of overt hyperthyroidism treated with radioactive iodine (RAI) and subclinical hyperthyroidism [56].

Mangement of thyrotoxicosis

The management of hyperthyroidism depends on the etiology, severity of the disease, patient's age, goiter size, concomitant diseases, and treatment preferences. The therapeutic objective is to rectify the hypermetabolic condition with as few adverse effects and as little hypothyroidism as possible. The mainstay of treatment is beta-blockade and anti-thyroid agents, propylthiouracil (PTU), and methimazole (MMI).

Beta-blockers are used for prompt control of adrenergic symptoms such as increased heart rate (sinus tachycardia); in fact, they are indicated in the rate control strategy of AF [57]. Propranolol is a non-selective beta-blocker and is the preferred drug as it has a direct effect on hypermetabolism and is used to control thyroid-induced tachyarrhythmias [1,6,58]. In contrast to other beta-blockers, propranolol can be used several times a day but is contraindicated in patients with pre-existing cardiac disease. However, Ca^+2^ channel blockers such as diltiazem or verapamil can be used in patients who cannot tolerate beta-blockers. Guidelines indicate using metoprolol, bisoprolol, carvedilol, or nebivolol in HF patients with decreased left ventricular ejection fraction, myocardial infarction, or as an antianginal therapy [59]. Palmieri et al. conducted a study to assess the effect of the beta-1 adrenergic blockade on myocardial contractility and total arterial stiffness in thyrotoxicosis. The study revealed that patients taking bisoprolol had normalization of total arterial stiffness, with decreased cardiovascular hyperkinesia, demonstrated as reduced heart rate [60].

Anti-thyroid drugs (PTU and MMI) are used in conjunction with beta-blockers. These drugs interfere with the organification of iodine, thus suppressing thyroid hormone synthesis. MMI cannot be used in pregnant patients because of its longer half-life and rare congenital abnormalities. On the other hand, PTU is safe and can be used in pregnancy. However, these anti-thyroid drugs are associated with remission. Irrespective of the regimen chosen, relapse can occur in up to 50% of patients who react initially [6]. In a randomized controlled experiment comparing MMI alone to MMI plus alpha-adrenergic blocking medication, patients receiving alpha-adrenergic blockers had lower heart rates, less shortness of breath and exhaustion, and improved "physical functioning" on the 36 questions short-form survey (SF-36) questionnaire after four weeks [61]. The other treatment modalities include RAI therapy and thyroidectomy.

Management of atrial fibrillation

Managing patients with AF and subclinical hyperthyroidism is aimed at preventing symptoms with a rate control strategy or rhythm control strategy and avoiding complications associated with AF such as HF, stroke, and others. Cardioversion has not been the primary priority since the stimulus to induce AF is still there at that moment, and a return to AF is possible. Thus, the essential goal of therapy was to control heart rate, which could be accomplished by combining anti-thyroid medications with routine pharmacologic therapy such as beta-blockers, digitalis, and diltiazem [57]. Initially, the patient should revert to a euthyroid state by using the drugs as mentioned earlier to maintain cardioversion and catheter ablation. Cardioversion may be used in individuals who still have AF after eight to ten weeks of remaining in a euthyroid state and using anticoagulation for at least three weeks [62]. After a year of diagnosis, a high risk of recurrence was noted because the chances of failed catheter ablation increase with time [63].

Although we have limited literature on who are good candidates for cardioversion, when thyroid hormone levels begin to fall due to anti-thyroid medication, AF spontaneously returns to sinus rhythm in many people. One previous research published in 1982 found that 62% of patients with AF returned to sinus rhythm during the first three to four months after thyrotoxicosis was treated, even without antiarrhythmic medication [64]. In catheter ablation, no clear consensus has been made on its efficacy. Ma et al. experimented with evaluating the clinical effectiveness of circumferential pulmonary vein ablation (CPVA) in hyperthyroid patients that revealed CPVA was a safe and viable therapeutic option for hyperthyroidism-related AF [65]. However, in a study by Machino et al., the efficacy of radiofrequency ablation with PV isolation was observed in hyperthyroid AF patients rendered euthyroid for three months compared to non-thyroid AF patients' recurrence of AF was the same as in the non-thyroid AF patients [66]. Therefore, ablation should be considered in patients with permanent, refractory AF despite the reversal of the euthyroid state.

AF and hyperthyroidism have been found to enhance the risk of thrombotic events independently. Anticoagulation plays a vital role in controlling thrombotic or embolic events [67]. However, a study by Chan et al. revealed that hyperthyroidism was not an independent risk factor for thrombosis in patients with AF. Therefore, anticoagulation should be done according to the CHA2DS2-VASC (congestive heart failure, hypertension, age ≥75 (doubled), diabetes, stroke (doubled), vascular disease, age 65 to 74 and sex category (female)) score [67,68]. Anticoagulation is recommended in case of a high-risk score (CHA2DS2-VASC equal to or more than 2). In a low-risk score, given the possibility of hypercoagulable stability, the continuation of subclinical hyperthyroidism and AF would argue for the use of anticoagulation. The summary of the included studies is given in Table 2.

**Table 2:** Summary of included studies establishing the risk of cardiac arrhythmias in hyperthyroidism TSH: thyroid-stimulating hormone/thyrotropin; T3: triiodothyronine; T4: thyroxine; ECG: electrocardiogram; HRV: heart rate variability; SVPD: premature supraventricular depolarization; SVT: supraventricular tachycardia; CVD: cardiovascular disease; AF: atrial fibrillation

References	Design	Cases	Population	Diagnostic criteria	Conclusions
Auer et al., 2001 [25]		23,638	Group 1: normal TSH, free T3, and T4 (22,300); Group 2: low TSH, high T3 and T4 (overt hyperthyroidism); Group 3: low TSH, low T3, T4 (Subclinical hyperthyroidism)	TSH, T3, and T4 levels	A low TSH concentration is associated with more than five-fold higher risk of atrial fibrillation.
Sawin et al., 1994 [22]	Cohort	2007	60 years or older who did not have AF	Serum TSH (low, normal, high values)	A low TSH serum concentration is associated with three-fold higher risk of AF in the elderly over subsequent decades.
Chen et al., 2006 [33]		32	Hyperthyroid Graves’ disease patients	ECG (HRV spectral analysis)	Hyperthyroid is characterized by increased sympathetic and decreased vagal modulation of heart.
Wustmann et al., 2008 [34]		28		Abnormal SVPD, number of SVT, heart rate variability	Hyperthyroidism is strongly correlated with increased supraventricular ectopic activity associated with AF.
Frost et al., 2004 [21]	Population-based study	40,628	Hyperthyroid patients	ECG	Male gender, increasing age, and prior CVD is associated with increased risk of AF.
Osman et al., 2004 [46]	Cohort	259	Overt hyperthyroid patients	24-hour Holter monitoring	A decrease of HRV and HRT in overt hyperthyroid patients that returned to normal with antithyroid therapy.
Brandt et al., 2011 [55]	Meta-analysis	31,138 and 400,000	Hyperthyroid patients and at-risk patients	TSH, T3, and T4	Mortality was increased by 20% in patients diagnosed with hyperthyroidism.
von Olshausen et al., 1989 [13]		37	Hyperthyroid patients	History, examination, ECG, echo, TFTs, chest x-ray and 24-hour Holter monitoring	Supraventricular arrhythmias were more common in hyperthyroid patients while ventricular arrhythmias were infrequent.

In conclusion, anticoagulation is beneficial, but it depends on the individual risk factors. Pharmacological rhythm control is often not suggested in patients with hyperthyroidism and AF since approximately two-thirds of individuals return to normal sinus rhythm 8-10 weeks after attaining euthyroid status [62]. Although, we can consider rhythm control in patients who continue to have AF after the euthyroid state. Drugs used for rhythm control include antiarrhythmic class 1A, 1C, and class 3 drugs. However, amiodarone may be used briefly during a thyroid storm to restore sinus rhythm or chronically in patients with AF resistant to rate control. While the use of amiodarone in AF is not yet well established, it causes hyperthyroidism as well as hypothyroidism due to the intrinsic effect of the drug. Type 1 amiodarone-induced hyperthyroidism (AIT) occurs in patients with underlying thyroid disease whereas Type 2 AIT is seen in people with normal thyroid glands [69].

Strengths and limitations

Although cardiac arrhythmias are an extensive and complex topic and have multiple etiologies, our review targets only hyperthyroidism as a risk factor and one of the causes of cardiac arrhythmias with no co-morbidities. However, this study does not address the other significant risk factors leading to cardiac arrhythmias and additional hyperthyroidism-related cardiovascular complications. Very few studies have collectively discussed hyperthyroid-induced atrial and ventricular arrhythmias; therefore, more studies need to be conducted to fill the knowledge gaps.

## Conclusions

From the studies discussed in this article, it can be seen that hyperthyroidism affects cardiovascular hemodynamics, predisposing a risk of cardiac arrhythmias. The most common arrhythmia described worldwide is AF, associated with increased mortality risk in the elderly. Sinus tachycardia, caused by sympathovagal dysfunction and increased intrinsic activity of the SA nodes, is also prevalent, whereas ventricular tachycardia and fibrillation are less common. The clinical implications of this review article are to identify the significance of thyroid-induced cardiac arrhythmias in a patient with thyrotoxicosis and improve the long-term prognosis and risk of thromboembolic events through appropriate treatment of hyperthyroidism with anti-thyroid medications and the control of heart rate by b-blockers which have proven to be very effective. Moreover, electrical or pharmacological cardioversion, catheter ablation, and anticoagulation can be considered in cases of AF. We feel our review article can serve to overcome challenges by providing a unique approach to the association between thyrotoxicosis and cardiac arrhythmias by emphasizing the profound effects of thyroid hormones on the cardiovascular system, the pathophysiology underlying tachyarrhythmias, and the management options. These concerns could be overcome by early recognition of hyperthyroidism with regular screening and ECG monitoring. At the same time, patients with AF should be evaluated for thyroid dysfunction by measuring TSH, T4, and T3 levels. Finally, more research studies may be required to help answer various issues about the management changes when co-morbid hyperthyroidism is present, and a more comprehensive approach is necessary.
